# Accuracy of the serum intestinal fatty-acid-binding protein for diagnosis of acute intestinal ischemia: a meta-analysis

**DOI:** 10.1038/srep34371

**Published:** 2016-09-29

**Authors:** Da-Li Sun, Yun-Yun Cen, Shu-Min Li, Wei-Ming Li, Qi-Ping Lu, Peng-Yuan Xu

**Affiliations:** 1 Department of Gastrointestinal Surgery, Second Affiliated Hospital of Kunming Medical University, Kunming 650101, China; 2 Research Center for Surgical Clinical Nutrition in Yun-Nan Province, Kunming 650101, China; 3 Department of General Surgery, Wuhan Clinical School of Southern Medical University/Wuhan General Hospital of Guangzhou Military Command, Wuhan 430070, China

## Abstract

Numerous studies have investigated the utility of serum intestinal fatty-acid binding protein (I-FABP) in differentiating acute intestinal ischemia from acute abdomen. However, the results remain controversial. The aim of this meta-analysis is to determine the overall accuracy of serum I-FABP in the diagnosis of acute intestinal ischemia. Publications addressing the accuracy of serum I-FABP in the diagnosis of ischemic bowel diseases were selected from databases. The values of true-positive (TP), true-negative (TN), false-positive (FP), and false-negative (FN) were extracted or calculated for each study. Pooled sensitivity, specificity, positive likelihood ratio (PLR), negative likelihood ratio (NLR), and diagnostic odds ratio (DOR) were calculated. The overall diagnostic performance was assessed using a summary receiver operating characteristic curve (SROC) and area under curve (AUC). Nine studies that collectively included 1246 patients met the eligible criteria. The pooled sensitivity, specificity, DOR, PLR, and NLR were 0.80 (95% CI: 0.72–0.86), 0.85 (95% CI: 0.73–0.93), 24 (95% CI: 9–65), 5.5 (95% CI: 2.8–10.8) and 0.23 (95% CI: 0.15–0.35), respectively. The AUC was 0.86 (95% CI: 0.83–0.89). The meta-analysis carried out in this report suggests that the I-FABP may be a useful diagnostic tool to confirm acute intestinal ischemia in acute abdomen, but better-designed trials are still required to confirm our findings.

Acute abdomen stands for a group of abdominal symptoms that rapidly worsen and require immediate treatment. Acute intestinal ischemia is a cause of severe acute abdomen with high rates of morbidity and mortality^1^. The causes are generally divided into two categories: vascular origin (mesenteric embolism/thrombosis, or non-occlusive mesenteric ischemia) or non-vascular causes such as strangulated intestinal obstruction^2^,^3^. Delayed diagnosis leads to intestinal necrosis and even multiple organ failure. Fast, accurate diagnosis is vital to improving the clinical outcomes of patients with acute intestinal ischemia. Even high-tech diagnostic equipment, such as computerized tomographic scanning (CT), can sometimes miss acute intestinal ischemia^4^,^5^. It is not easy, even for experienced clinicians, to identify patients who are at risk of acute intestinal ischemia among patients presenting with acute abdomen. In recent years, many circulating biomarkers including intestinal fatty-acid binding protein (I-FABP), glutathione S-transferase (GST), D-lactate, diamine oxidase (DOA), and citrulline have been investigated as potentially effective biomarkers for acute intestinal ischemia^6^,^7^,^8^.

I-FABP is a small (14–15 kDa), cytosolic, water-soluble protein that is abundant in the small intestinal mucosa. It accounts for approximately 2% of the cytosolic proteins^9^. An increasing number of studies have shown that the serum I-FABP level is elevated in patients with intestinal ischemia^10^,^11^,^12^,^13^,^14^,^15^,^16^,^17^,^18^,^19^,^20^. However, due to the varying degree of diagnostic accuracy of I-FABP reported in different studies, serum I-FABP has not yet been used in a clinical setting. Systematic analysis of these data may be valuable to finally confirm the application of serum I-FABP as a potential biomarker for acute intestinal ischemia. Therefore, we performed a meta-analysis to summarize the literature in the databases, on the overall accuracy of serum I-FABP for differentiating acute intestinal ischemia from acute abdomen.

## Methods

### Search strategy

This meta-analysis was performed and reported according to the guideline set out for diagnostic studies^21^. We searched for diagnostic studies published in PubMed, Medline (Ovid), Cochrane Library, Web of Science, and China National Knowledge Infrastructure (CNKI) up to December 26, 2015. No language limits were applied. Searching was limited to publications with clinical trials. Terms for searching, both free text and medical subject headings (MeSH), included the following alternatives for intestine or mesentery: “intestines”, “intestinal”, “bowel”, “gut”, “mesentery”, “mesaraic”, “mesenteric”; individually with each of the following variations on ischemia: “ischemia”, “ischemic”, “reperfusion.” These terms were searched alone and in combination. The results were then combined using the set operator “AND” with studies identified by varied diagnostic terms: “sensitivity”, “specificity”, “false positive”, “false negative”, “accuracy”, “predictive value of tests”, “likelihood ratio”, “reference values”, “roc analysis” and “intestinal fatty acid-binding protein” or “I-FABP.” Additional relevant studies were included by manually searching the references of identified articles and relevant review articles.

### Study selection and extraction

Eligible studies were selected according to the following inclusion criteria: (1) studies evaluated I-FABP in the differential diagnosis of intestinal ischemia, (2) each study consisted of more than 10 blood specimens, and (3) studies had sufficient data to construct a diagnostic 2 × 2 table. Exclusion criteria: (1) studies did not report sensitivity or specificity of I-FABP; (2) studies did not report the definition of reference standards for diagnosis of intestinal ischemia; (3) studies included methodological mistakes. Reference standards for diagnosis of intestinal ischemia were defined as patients with clinical symptoms such as acute abdominal pain, frequent vomiting, decreases in flatus or defecation, distension, or no bowel sounds, with evidence of histopathological or radiological examination, or operative findings.

The studies were imported into a bibliographic database to automatically exclude duplicates. Two independent reviewers (Y.Y.C. and S.M.L.) judged the eligibility of the studies while screening the citations. Any disagreements between eligibility or data extraction were corrected by consensus. Consensus was reached through discussion and where this could not be reached a third researcher arbitrated. The first author’s name, gender, publication year, country, I-FABP methods of detection, cutoff value, sensitivity, and specificity were retrieved.

### Methodology quality appraisal

Two investigators independently assessed studies selected for inclusion in the study for methodological quality using QUADAS (Quality Assessment of Diagnostic Accuracy Studies included in systematic reviews)^22^. QUADAS is an evidence-based quality assessment tool for use in systematic reviews of diagnostic accuracy studies, including 14 items (maximum score, 14)^22^ (Table S1).

### Data analysis

The methods for the diagnostic accuracy of meta-analyses were the same as those used by Leeflang MM, *et al*.^21^ and Jones CM, *et al*.^23^. The pooled sensitivity, specificity, PLR, NLR, positive predicted value, negative predicted value, DOR and corresponding 95% confidence intervals (95% CI) were calculated by the accuracy data (TP, TN, FP and FN) extracted from each of the included studies. Heterogeneity due to the threshold effect (differences in sensitivity and specificity occurring because of different cut-offs used in different studies to define a positive test result) was investigated using the Spearman correlation coefficient using Meta-Disc 1.4 for Windows (XI Cochrane Colloquium, Barcelona, Spain). A strong positive correlation was observed (*P* < 0.05), suggesting a significant threshold effect. The heterogeneity caused by non-threshold effect was measured by the chi-square-based Q test and the inconsistency index I^2^. A significant Q test (p < 0.05 or I^2^  > 50%) indicated heterogeneity among studies. In the case of significant heterogeneity, the DerSimonian Laird method was used to calculate the estimates^24^. Meta-regression was performed to detect the source of heterogeneity within the included studies. Bias in publication was tested by using Deeks’ funnel plots^25^. A *P* value < 0.05 indicated the presence of publication bias. In addition to the analysis of the threshold effect, other analyses were performed using the STATA software (version 12.0, Stata Corporation, College Station, TX, USA).

## Results

### Study selection

**Figure 1:**
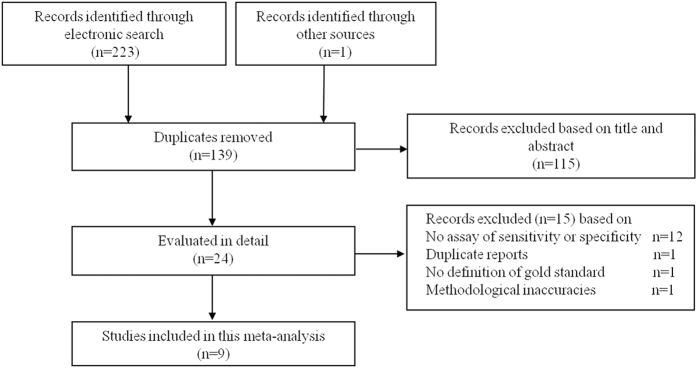
Summary of evidence search and selection.

Our study search initiated 224 potentially relevant studies. We excluded 89 duplicates and 115 papers based on title and abstract screening. Twenty four papers were retrieved in full text. Fifteen papers were excluded, and only nine papers met the eligibility criteria (Fig. 1). Among the 15 excluded papers, 12 studies did not report sensitivity or specificity of I-FABP^9^,^18^,^20^,^26^,^27^,^28^,^29^,^30^,^31^,^32^,^33^,^34^, one study was a duplicate^35^, one study did not report the definition of reference standard for diagnosis^36^ and one study had methodological inaccuracies^37^.

### Characteristics of studies of the meta-analysis

Basic characteristics of studies were shown in Table 1 from the literature that was published between 2006 and 2015. Overall, among 9 such studies of which only 1 study was carried out in USA^16^, 2 studies were from Netherlands^13^,^19^ and the other 6 studies were all performed in Asia^10^,^11^,^12^,^14^,^15^,^17^. 1246 patients with suspected acute intestinal ischemia were included in this meta-analysis. There were several types of acute intestinal ischemia, 2 studies reported strangulated small bowel obstruction^14^,^16^. 1 study did not describe the type of acute intestinal ischemia^13^, 1 study reported vascular intestinal ischemia^11^, 1 study included intestinal necrosis^19^, and 4 studies included mixing types^10^,^12^,^15^,^17^. Reference standards for diagnosis such as histopathological examination, operative findings, and radiological findings were applied to detection of acute intestinal ischemia (Table 1). QUADAS score summaries were presented in Table 1 and Table S1.

### Accuracy of serum I-FABP biomarker for the detection of acute intestinal ischemia

**Figure 2:**
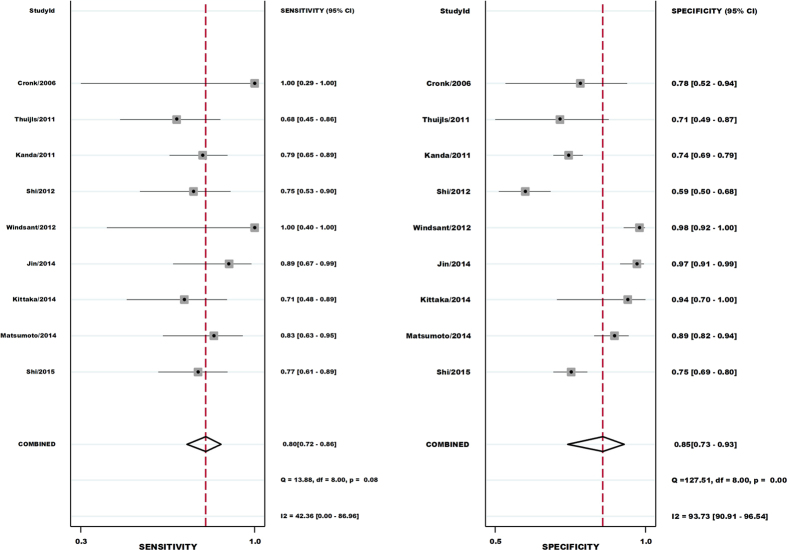
Forest plot of sensitivity and specificity of serum I-FABP.

**Figure 3:**
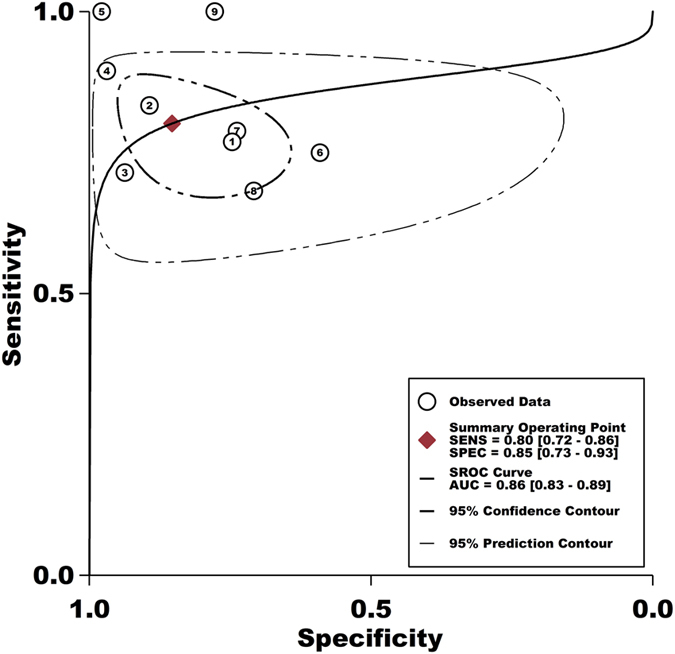
The summary operative receiver characteristic curve indicated high diagnostic accuracy.

The threshold effect was caused by differences in sensitivity and specificity. Spearman correlation coefficient and *P* value were applied to assessing the threshold effect. The Spearman correlation coefficient was −0.683 and the *P* value was 0.042, suggesting that no enough evidence supported heterogeneity from threshold effect. The pooled sensitivity and specificity of serum I-FABP for the detection of acute intestinal ischemia were 0.80 (95% CI: 0.72–0.86) and 0.85 (95% CI: 0.73–0.93), respectively (Fig. 2 and Table S2). The PLR and NLR of serum I-FABP were 5.5 (95% CI: 2.8–10.8) and 0.23 (95% CI: 0.15–0.35), respectively (Table S2). The DOR was 24 (95% CI: 9–65) (Table S2). Figure 3 shows the SROC with AUC to be 0.86 (95% CI: 0.83–0.89), indicating that the overall accuracy was high.

### Meta-regression analysis

As shown in the forest plots of accuracy data (specificity, PLR, NLR, and DOR) in Fig. 2, S1, and S2, heterogeneity was significant. To explore the possible reasons for the heterogeneity, a meta-regression analysis for continuous covariates (sample size, cutoff value, and QUADAS scores) and a binary covariate (reference standard for diagnosis (histopathological or non-histopathological)) was performed. However, none of the above covariates were found to be a significant source of heterogeneity (Table 2 and Figure S3).

### Sensitivity analysis and publication bias

Sensitivity analyses were performed based on studies carried out after 2005 and studies that used enzyme-linked immunosorbent assays to detect I-FABP, and the pooled results were similar to the results of the overall analysis (Table S2). Publication bias was tested using the Deek’s funnel plot. As shown in Figure S4, there was no significant publication bias (*P* = 0.260 (>0.05)).

## Discussion

A feasible, reliable, and minimally invasive approach to detect acute intestinal ischemia has been a limiting factor in clinical practice. Although acute intestinal ischemia can be confirmed by histopathological examination or angiography, identifying acute intestinal ischemia from patients with suspected acute intestinal ischemia, especially on an emergency basis, is often difficult, and sometimes impossible. As a more feasible and less invasive alternative, I-FABP has attracted more and more attention of researchers^9^,^10^,^11^,^12^,^13^,^14^,^15^,^16^,^17^,^18^,^19^,^20^,^26^,^27^,^28^,^29^,^30^,^31^,^32^,^33^,^34^,^35^,^36^,^37^. However, the accuracy of serum I-FABP for diagnosis has been reported to be variable.

This is the first meta-analysis of the accuracy of serum I-FABP for diagnosis of acute intestinal ischemia. The pooled sensitivity for serum I-FABP was 0.80 while the pooled specificity was 0.85. An AUC value of 0.86 and a DOR value of 24 indicated a high accuracy in the diagnosis of acute intestinal ischemia.

Heterogeneity is a potential obstacle to interpretation of results for any meta-analysis. The I^2^ test for the pooled specificity, DLR, PLR, and DOR indicated that the significant heterogeneity between the studies was observed (*P* < 0.05). Threshold effect is one of the major causes of heterogeneity in diagnostic studies due to variable cut-offs. Most of the studies used enzyme-linked immunosorbent assays to detect I-FABP. Of these, 8 covered intestinal ischemia of different types and levels of severity, which determined the serum I-FABP level. ELISA kits with different levels of performance, different detecting instruments, and different operation processes might also have affected the I-FABP value^38^,^39^. For these reasons, Spearman correlation was performed to analyze the threshold effect. A negative correlation (Spearman correlation coefficient was −0.683 and the *P* value was 0.042) did not support that threshold effect led to heterogeneity. A meta-regression analysis was undertaken to detect other possible reasons for heterogeneity, including QUADAS scores, sample size, cutoff value and reference standard for diagnosis. Unfortunately, none of the analyzed covariates were the source of heterogeneity.

Sensitivity analyses were performed to improve the homogeneity of the results. Even if enzyme-linked immunosorbent assays had been used to test I-FABP in all the analyzed studies spanning more than a decade, the experimental conditions are still significantly different, which would affect the results of each individual study. When a study that was undertaken in 2002 was excluded^16^, the sensitivity analysis results were similar to the overall results with slightly increased specificity. There was no evidence that the experimental conditions have impact on the value of I-FABP. In addition, different methods of detection may influence the accuracy of I-FABP for diagnosis of acute intestinal ischemia. So we performed another sensitivity analysis by focusing on studies that used enzyme-linked immunosorbent assays to detect I-FABP^10^,^11^,^12^,^13^,^14^,^15^,^16^,^17^. The summary results showed a modest decrease of sensitivity and specificity, compared with the overall results, but additional studies on this topic are needed.

The causes of acute intestinal ischemia include vascular and non-vascular. One study indicated that I-FABP is more valuable in the diagnosis of vascular intestinal ischemia than in the diagnosis of non-vascular intestinal ischemia^11^. If the subgroup analysis can be carried out, the results can be further verified. Unfortunately, only 4 studies that clearly reported the sensitivity and specificity of different types of acute intestinal ischemia were available^11^,^14^,^16^,^19^. The sample size was not large enough to support the subgroup analysis.

Our review has some limitations. First, more than half of the studies analyzed were small, which may cause overestimation of the true diagnostic accuracy of I-FABP in the diagnosis of acute intestinal ischemia. To assess the effects of sample size, meta-regression was carried out, and results suggested that the pooled results were stable and not affected by bias. However, large-scale, high-quality studies are still needed to assess diagnostic accuracy. Most of the included studies did not differentiate the type or severity of intestinal ischemia. Different cutoff values were used in each study, which made it difficult to determine the optimized cutoff value. Third, limitations also exist in the reference standard for diagnosis of acute intestinal ischemia. Histopathological examination is an ideal reference standard, but it is difficult or impossible to obtain pathologic specimen in some disease states. So some included studies used radiological examination to diagnose acute intestinal ischemia. Even if meta-regression was performed to assess the effect of different reference standards, some analytical studies used mixed reference standards (including histopathological examination, radiological examination, and operating findings). It also leads to overestimation of diagnostic accuracy. For these reasons, future work should focus on comparing the diagnostic accuracy of I-FABP using histopathological examination to minimize the current weakness.

Despite these limitations, these data suggest that serum I-FABP appears to be of adequate diagnostic value with respect to accurately differentiating acute intestinal ischemia from suspected acute intestinal ischemia. However, the results should be interpreted in parallel with histopathological examination and clinical findings to confirm the diagnosis. Further study with larger sample size should be warranted for clarification.

## Additional Information

**How to cite this article**: Sun, D.-L. *et al*. Accuracy of the serum intestinal fatty-acid-binding protein for diagnosis of acute intestinal ischemia: a meta-analysis. *Sci. Rep.* **6**, 34371; doi: 10.1038/srep34371 (2016).

## Supplementary Material

Supplementary Information

## Figures and Tables

**Table 1:** Characteristics of the included studies.

Author	Year	Country	Types of acute intestinal ischemia	Reference standard	Sample size	Male	I-FABP methods of detection	Cut-off value (ng/ml)	Major findings	QUADAS score
Shi H^10^	2015	China	Bowel strangulation, mesenteric infarction, ischemic colitis	histopathological examination	272	180	Enzyme-linked immunosorbent assays	82.4	Serum I-FABP and D-lactate can improve the diagnosis of intestinal ischemia in patients with acute abdomen	12
Matsumoto S^11^	2014	Japan	Non-occlusive ischaemia, mesenteric arterial occlusion	radiological, surgical and histopathological examination	146	98	Enzyme-linked immunosorbent assays	9.1	I-FABP shows promise for detecting vascular ischaemia	12
Kittaka H^14^	2014	Japan	Strangulated small bowel obstruction	CT findings and operative findings	37	17	Enzyme-linked immunosorbent assays	6.5	The I-FABP level is a useful marker for discriminating strangulated small bowel obstruction from small bowel obstruction	13
Jin H^15^	2014	China	Mesenteric infarction, focal colonic ischemia	histopathological examination or operative findings	116	73	Enzyme-linked immunosorbent assays	101.1	Serum I-FABP is a useful marker for diagnosis of small bowel ischemia	9
Vermeulen Windsant IC^19^	2012	Netherlands	Intestinal necrosis after aortic surgery	histopathological examination	96	70	Synthetic regional peptides and a recombinant I-FABP assays	5.787	Analysis of plasma IFABP levels is of additional value to other current plasma markers in the diagnosis of intestinal necrosis	12
Shi H^18^	2012	China	Mesenteric infarction, ischemic colitis	radiological, surgical and histopathological examination	151	105	Enzyme-linked immunosorbent assays	87.52	I-FABP is a potentially useful for discriminating intestinal ischemia from acute abdomen	11
Kanda T^12^	2011	Japan	Strangulated bowel obstruction, mesenteric infarction, perforation of the gastrointestinal tract, ischemic enterocolitis	operative findings	361	218	Enzyme-linked immunosorbent assays	3.1	Serum I-FABP measurement is a non-invasive method that is potentially useful for diagnosis of small bowel ischemia	13
Thuijls G^13^	2011	Netherlands	Acute intestinal ischemia ( did not describe types)	histopathological examination or determined by consensus among 2 of the authors	46	18	Enzyme-linked immunosorbent assays	0.268	Serum I-FABP can improve early diagnosis of intestinal ischemia	11
Cronk DR^16^	2006	USA	Strangulated small bowel obstruction	operative findings	21	NA	Enzyme linked immunosorbent assays	0.1	I-FABP is a sensitive marker for ischemia in mechanical small bowel obstruction	12

NA: No reports.

**Table 2:** Meta-regression results.

	Number of studies	Sensitivity (95% CI)	Specificity (95% CI)	LRT χ^2^	I^2^	P value
QUADAS scores	9	0.79 (0.69–0.86)	0.82 (0.63–0.92)	0.67	0	0.72
Sample size	9	0.79 (0.71–0.86)	0.86 (0.75–0.92)	1.61	0	0.45
Cutoff value	9	0.80 (0.72–0.86)	0.85 (0.74–0.92)	0.22	0	0.90
Reference standard for diagnosis						
Histopathological	6	0.81 (0.72–0.89)	0.87 (0.77–0.97)	0.24	0	0.89
Non-histopathological	3	0.79 (0.67–0.90)	0.82 (0.62–1.00)			

LRT: likelihood ratio test.

## References

[b1] Acosta S. Epidemiology of mesenteric vascular disease: clinical implications. Semin Vasc Surg 23, 4–8 (2010). 20298944 10.1053/j.semvascsurg.2009.12.001

[b2] Menke J. Diagnostic accuracy of multidetector CT in acute mesenteric ischemia: systematic review and meta-analysis. Radiology 256, 93–101 (2010). 20574087 10.1148/radiol.10091938

[b3] Furukawa A. . CT diagnosis of acute mesenteric ischemia from various causes. AJR Am J Roentgenol 192, 408–416 (2009). 19155403 10.2214/AJR.08.1138

[b4] Angelelli G. . Acute bowel ischemia: CT findings. Eur J Radiol 50, 37–47 (2004). 15093234 10.1016/j.ejrad.2003.11.013

[b5] Kozuch P. L. . Review article: diagnosis and management of mesenteric ischemia with an emphasis on pharmacotherapy. Aliment Pharmacol Ther 21, 201–215 (2005). 15691294 10.1111/j.1365-2036.2005.02269.x

[b6] Evennett N. J. . Systematic review and pooled estimates for the diagnostic accuracy of serological markers for intestinal ischemia. World J Surg 33, 1374–1383 (2009). 19424744 10.1007/s00268-009-0074-7

[b7] Crenn P. . Citrulline as a biomarker of intestinal failure due to enterocyte mass reduction. Clin Nutr 27, 328–339 (2008). 18440672 10.1016/j.clnu.2008.02.005

[b8] Cakmaz R. . A combination of plasma DAO and citrulline levels as a potential marker for acute mesenteric ischemia. Libyan J Med 8, 1–6 (2013). 23534825 10.3402/ljm.v8i0.20596 PMC3609998

[b9] Pelsers M. M. . Intestinal-type and liver-type fatty acid-binding protein in the intestine. Tissue distribution and clinical utility. Clin Biochem 36, 529–535 (2003). 14563446 10.1016/s0009-9120(03)00096-1

[b10] Shi H. . The role of serum intestinal fatty acid binding protein levels and D-lactate levels in the diagnosis of acute intestinal ischemia. Clin Res Hepatol Gastroenterol 39, 373–378 (2015). 25683524 10.1016/j.clinre.2014.12.005

[b11] Matsumoto S. . Diagnostic performance of plasma biomarkers in patients with acute intestinal ischaemia. BJS 101, 232–238 (2014). 10.1002/bjs.9331 24402763

[b12] Kanda T. . Diagnosis of ischemic small bowel disease by measurement of serum intestinal fatty acid-binding protein in patients with acute abdomen: a multicenter, observer-blinded validation study. J Gastroenterol 46, 492–500 (2011). 21298292 10.1007/s00535-011-0373-2

[b13] Thuijls G. . Early diagnosis of intestinal ischemia using urinary and plasma fatty acid binding proteins. Ann Surg 253, 302–308 (2011). 10.1097/SLA.0b013e318207a767 21245670

[b14] Kittaka H. . Usefulness of intestinal fatty acid-binding protein in predicting strangulated small bowel obstruction. PLoS One 13, e99915 (2014). 24926782 10.1371/journal.pone.0099915 PMC4057439

[b15] Jin H. Value of intestinal fatty acid binding protein in diagnose of ischemic bowel disease. Chin J Immuno (Chinese) 30, 531–533 (2014).

[b16] Cronk D. R. . Intestinal fatty acid binding protein (I-FABP) for the detection of strangulated mechanical small bowel obstruction. Curr Surg 63, 322–325 (2006). 16971202 10.1016/j.cursur.2006.05.006

[b17] Shi H. . The value of serum intestinal fatty acid blinding protein measurement in discriminating intestinal ischemia in patients with acute abdomen. Chin J Intern Med (Chinese) 51, 690–693 (2012). 23158918

[b18] Mensink P. B. . Transient postprandial ischemia is associated with increased intestinal fatty acid binding protein in patients with chronic gastrointestinal ischemia. Eur J Gastroenterol Hepatol 21, 278–282 (2009). 19279473 10.1097/MEG.0b013e32832183a7

[b19] Vermeulen Windsant I. C. . Circulating intestinal fatty acid-binding protein as an early marker of intestinal necrosis after aortic surgery: a prospective observational cohort study. Ann Surg 255, 796–803 (2012). 22367448 10.1097/SLA.0b013e31824b1e16

[b20] van der Voort P. H. . Can serum L-lactate, D-lactate, creatine kinase and I-FABP be used as diagnostic markers in critically ill patients suspected for bowel ischemia. BMC Anesthesiol 14, 111 (2014). 25844063 10.1186/1471-2253-14-111 PMC4384375

[b21] Leeflang M. M. . Systematic reviews of diagnostic test accuracy. Ann Intern Med 149, 889–897 (2008). 19075208 10.7326/0003-4819-149-12-200812160-00008 PMC2956514

[b22] Whiting P. . The development of QUADAS: a tool for the quality assessment of studies of diagnostic accuracy included in systematic reviews. BMC Med Res Methodol 3, 25 (2003). 14606960 10.1186/1471-2288-3-25 PMC305345

[b23] Jones C. M. . Diagnostic accuracy meta-analysis: a review of the basic principles of interpretation and application. Int J Cardiol 140, 138–144 (2010). 19592119 10.1016/j.ijcard.2009.05.063

[b24] Higgins J. P. . Measuring inconsistency in meta-analyses. BMJ 327, 557–560 (2003). 12958120 10.1136/bmj.327.7414.557 PMC192859

[b25] Deeks J. J. . The performance of tests of publication bias and other sample size effects in systematic reviews of diagnostic test accuracy was assessed. J Clin Epidemiol 58, 882–893 (2005). 16085191 10.1016/j.jclinepi.2005.01.016

[b26] Block T. . Diagnostic accuracy of plasma biomarkers for intestinal ischaemia. Scand J Clin Lab Invest 68, 242–248 (2008). 17934974 10.1080/00365510701646264

[b27] Kanda T. . Intestinal fatty acid-binding protein is a useful diagnostic marker for mesenteric infarction in humans. Gastroenterology 110, 339–343 (1996). 8566578 10.1053/gast.1996.v110.pm8566578

[b28] Hu K. Q. . Change of plasma intestinal fatty acid-binding protein and liver fatty acid-binding protein in children with intestinal ischemia. J Clin Pedia Surg (Chinese) 9, 95–97 (2010).

[b29] Hu K. Q. . The value of serum intestinal fatty acid blinding protein measurement in discriminating intestinal ischemia in children. Shanxi Med J (Chinese) 39, 418–419 (2010).

[b30] Yan W. L. . Diagnostic value of plasma intestinal fatty acid-binding protein and creatine kinase in patients subjected with intestinal obstruction. Zhongguo Zhong Xi Yi Jie He Za Zhi (Chinese) 19, 624–626 (2013).

[b31] Li L. & Liang H. F. Application of IFABP, LFABP detection technology in children with intestinal ischemia. Chin J Pharma Econom (Chinese) 1, 64–66 (2012).

[b32] Jin H. The relationship between intestinal fatty acid binding protein and the severity and prognosis of ischemic bowel disease. Zhe Jiang Lin Chuang Yi Xue (Chinese) 16, 371–372 (2014).

[b33] Shen D. H. . The value of serum intestinal fatty acid blinding protein in intestinal diseases. Shi Yong Yi Xue Za Zhi (Chinese) 21, 2535–2536 (2005).

[b34] van Noord D. . Serum markers and intestinal mucosal injury in chronic gastrointestinal ischemia. Dig Dis Sci 56, 506–512 (2011). 20628816 10.1007/s10620-010-1303-5 PMC3029832

[b35] Wang H. . The diagnosis value of combination of I-FABP, D-LAC, Fg in acute ischemic enteritis. Lin Chuang He Li Yong Yao (Chinese) 7, 144–145 (2014).

[b36] Zheng L. . The value of I-FABP measurement in the diagnosis of intestinal ischemic in patients with acute intestinal obstruction. Int J Lab Med (Chinese) 35, 410–411 (2014).

[b37] Wang H. . The diagnosing value of I-FABP, D-LAC in ischemic enteritis. Lin Chuang He Li Yong Yao (Chinese) 7, 112–114 (2014).

[b38] Riffelmann M. . Performance of commercial enzyme-linked immunosorbent assays for detection of antibodies to Bordetella pertussis. J Clin Microbiol 48, 4459–4463 (2010). 20943873 10.1128/JCM.01371-10 PMC3008456

[b39] van de Wouw B. A. . Comparison of three commercially available enzyme-linked immunosorbent assays and biopsy-dependentdiagnosis for detecting Helicobacter pylori infection. J Clin Microbiol 34(1), 94–97 (1996). 8748281 10.1128/jcm.34.1.94-97.1996 PMC228738

