# Supplementary material for: Accuracy of the serum intestinal fatty-acid-binding protein for diagnosis of acute intestinal ischemia: a meta-analysis
Source: Sci Rep. 2016 Sep 29;6:34371. doi: 10.1038/srep34371 (PMC5041130; doi:10.1038/srep34371)
Supplement: Supplementary Information [file srep34371-s1.pdf]

Accuracy of the serum intestinal fatty-acid-binding protein for diagnosis of acute intestinal ischemia: a meta-analysis

Da-Li Sun, Yun-Yun Cen, Shu-Min Li, Wei-Ming Li, Qi-Ping Lu, Peng-Yuan Xu

Supplementary table and figure legends

Supplementary tables

Table S1 Summary of study quality assessment
Table S2 Summary of overall analysis and sensitivity analysis

Supplementary figures

Figure S1 Forest plots of PLR and NLR

Figure S2 Forest plots of DOR

Figure S3 Meta regression

Figure S4 Deek's funnel plot showed no significant publication bias

Figure S5 Fagan plot presents the clinical utility of I-FABP

Accuracy of the serum intestinal fatty-acid-binding protein for diagnosis of acute intestinal ischemia: a meta-analysis

Da-Li Sun, Yun-Yun Cen, Shu-Min Li, Wei-Ming Li, Qi-Ping Lu, Peng-Yuan Xu

Table S1. Summary of quality assessment of studies

Author	Year	Was the spectrum of patients representative of the patients who will receive the test in practice?	Were selection criteria clearly described?	Was the execution of the index test described in sufficient detail to permit replication of the test?	Were the index test results interpreted without knowledge of the results of the reference standard?	Is the reference standard likely to correctly classify the target condition?	Were the reference standard results interpreted without knowledge of the results of the index test?	Is the time period between reference standard and index test short enough to be reasonably sure that the target condition did not change between the two tests?	Did the whole sample or a random selection of the sample, receive verification using a reference standard of diagnosis?	Did patients receive the same reference standard regardless of the index test result?	Was the reference standard independent of the index test	Was the execution of the reference standard described in sufficient detail to permit its replication?	Were the same clinical data available when test results were interpreted as would be available when the test is used in practice?	Were uninterpretable/ intermediate test results reported?	Were withdrawals from the study explained?	Score
Shi H [10]	2015	Yes	Yes	Yes	Yes	Yes	Yes	Yes	No	No	Yes	Yes	Yes	Yes	Yes	12
Matsumoto S [11]	2014	Yes	Yes	Yes	Yes	Yes	Yes	Yes	No	No	Yes	Yes	Yes	Yes	Yes	12
Kittaka H [14]	2014	Yes	Yes	Yes	Yes	Unclear	Yes	Yes	Yes	Yes	Yes	Yes	Yes	Yes	Yes	13
Jin H [15]	2014	Yes	Yes	Unclear	Unclear	Yes	Unclear	Yes	No	No	Yes	Yes	Yes	Yes	Yes	9
Vermeulen Windsant IC [19]	2012	Yes	Yes	Yes	Yes	Yes	Unclear	Yes	Yes	No	Yes	Yes	Yes	Yes	Yes	12
Shi H [18]	2012	Yes	Yes	Yes	Yes	Yes	Unclear	Yes	No	No	Yes	Yes	Yes	Yes	Yes	11
Kanda	2011	Yes	Yes	Yes	Yes	Yes	Yes	Yes	Yes	No	Yes	Yes	Yes	Yes	Yes	13

Accuracy of the serum intestinal fatty-acid-binding protein for diagnosis of acute intestinal ischemia: a meta-analysis

Da-Li Sun, Yun-Yun Cen, Shu-Min Li, Wei-Ming Li, Qi-Ping Lu, Peng-Yuan Xu

T [12]																
Thuijls G [13]	2011	Yes	Yes	Yes	Yes	Unclear	Yes	Yes	No	No	Yes	Yes	Yes	Yes	Yes	11
Cronk DR [16]	2006	Yes	Yes	Yes	Yes	Yes	Unclear	Yes	Yes	No	Yes	Yes	Yes	Yes	Yes	12

Total QUADAS score is out of 14. If the answer was “yes” to ten or more of the criteria listed for high quality, if the answer was “yes” to less than ten of the criteria listed for lower quality.

Accuracy of the serum intestinal fatty-acid-binding protein for diagnosis of acute intestinal ischemia: a meta-analysis

Da-Li Sun, Yun-Yun Cen, Shu-Min Li, Wei-Ming Li, Qi-Ping Lu, Peng-Yuan Xu

Table S2 Summary of overall analysis and sensitivity analysis

Variables	Number of studies	Sensitivity (95% CI)	Specificity (95% CI)	DOR (95% CI)	PLR (95% CI)	NLR (95% CI)	AUC
Overall analysis	9	0.80(0.72–0.86)	0.85(0.73–0.93)	24(9–65)	5.5(2.8–10.8)	0.23(0.15–0.35)	0.86(0.83–0.89)
Researches carried out after 2005	8	0.80(0.72–0.86)	0.86(0.73–0.93)	25(8–75)	5.7(2.7–12.4)	0.23(0.15–0.36)	0.86(0.83–0.89)
Studies used ELISA to detect I-FABP	8	0.79(0.72–0.84)	0.82(0.70–0.90)	17(7–40)	4.3(2.5–7.6)	0.26(0.18–0.37)	0.84(0.80–0.87)

DOR: diagnostic odds ratio; PLR: positive likelihood ratio; NLR negative likelihood ratio; AUC: area under curve; ELISA: enzyme-linked immunosorbent assays.

Accuracy of the serum intestinal fatty-acid-binding protein for diagnosis of acute intestinal ischemia: a meta-analysis

Da-Li Sun, Yun-Yun Cen, Shu-Min Li, Wei-Ming Li, Qi-Ping Lu, Peng-Yuan Xu

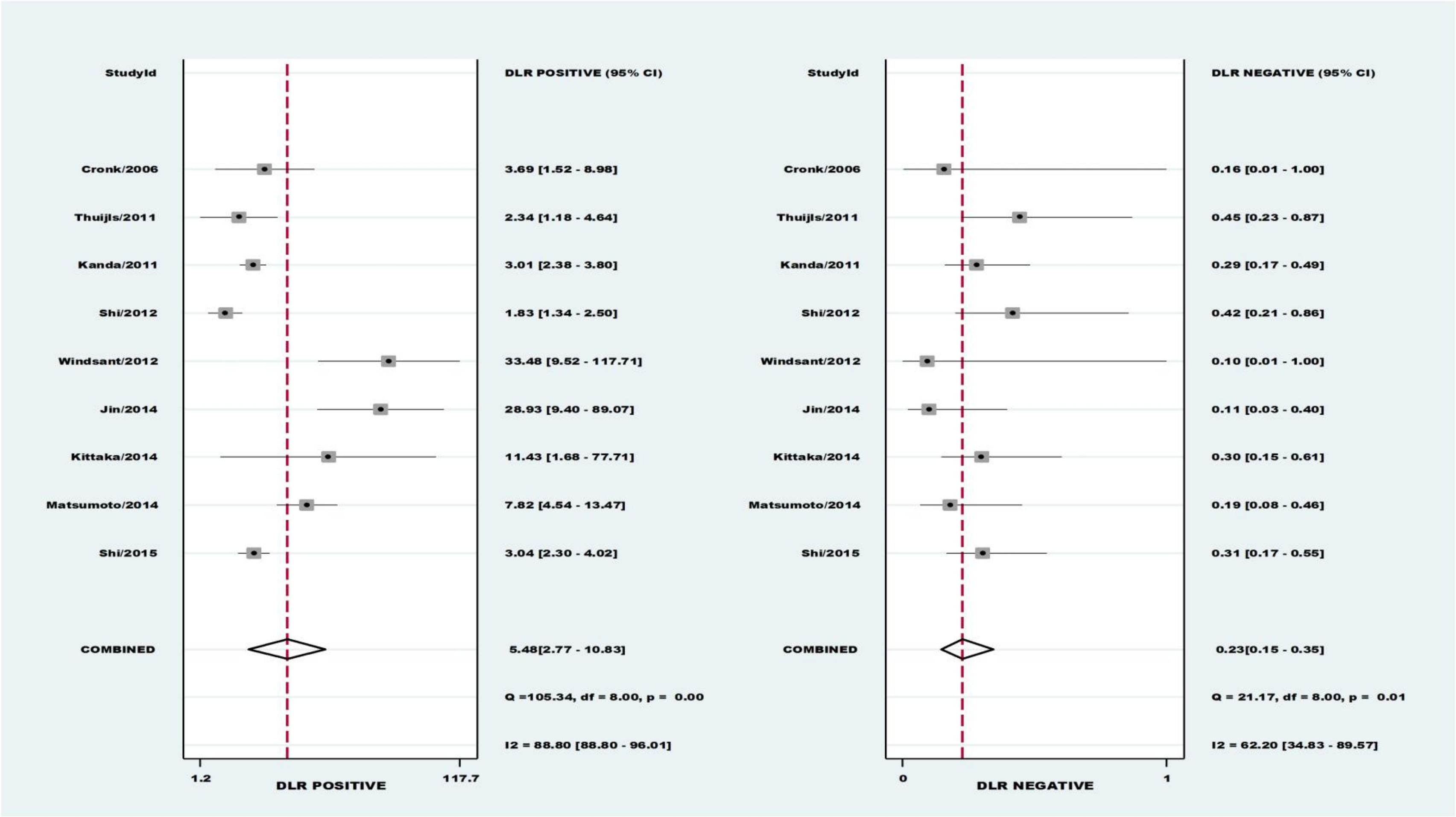

Figure S1. Forest plots of PLR and NLR

Accuracy of the serum intestinal fatty-acid-binding protein for diagnosis of acute intestinal ischemia: a meta-analysis

Da-Li Sun, Yun-Yun Cen, Shu-Min Li, Wei-Ming Li, Qi-Ping Lu, Peng-Yuan Xu

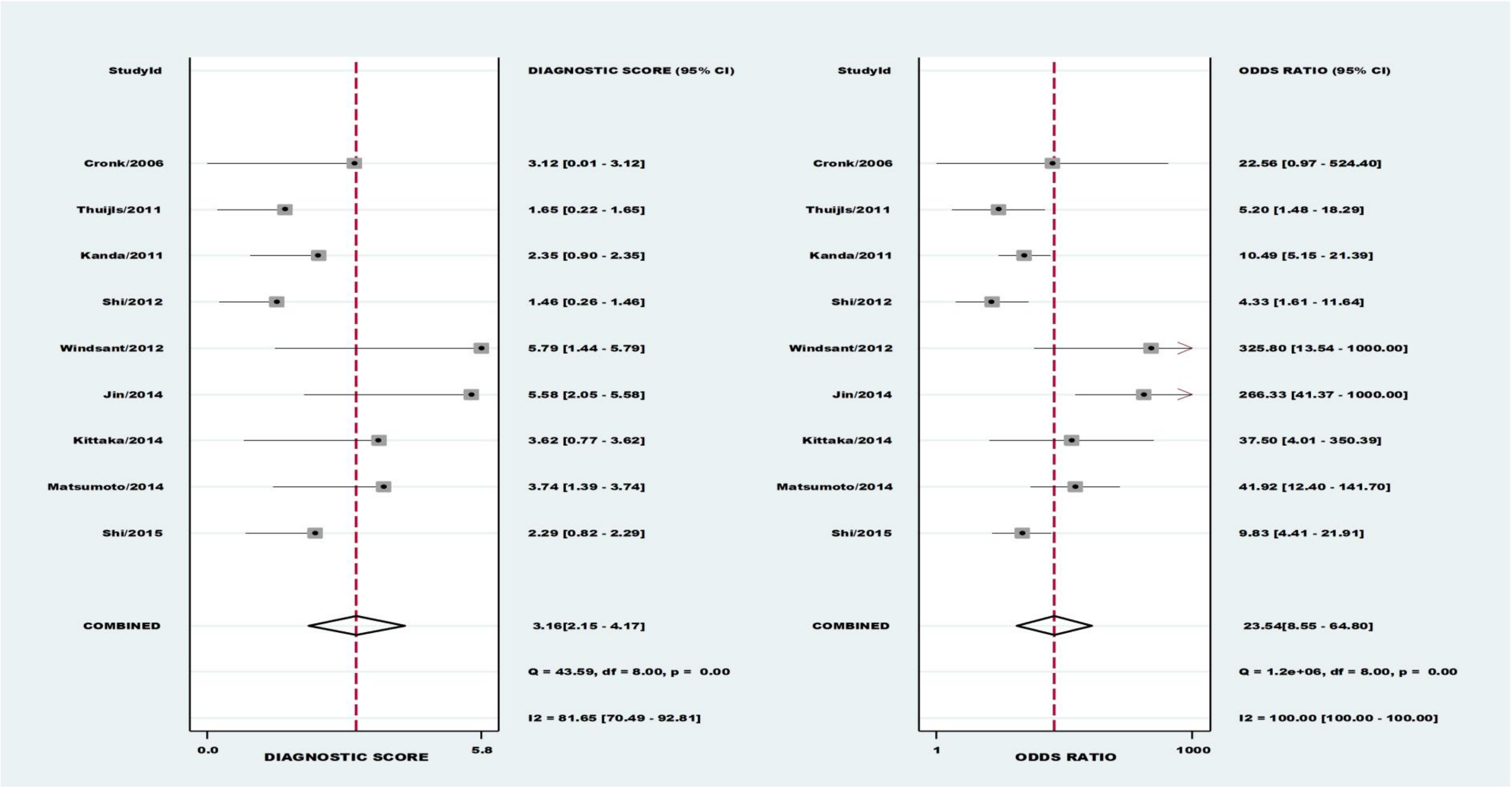

Figure S2. Forest plots of DOR

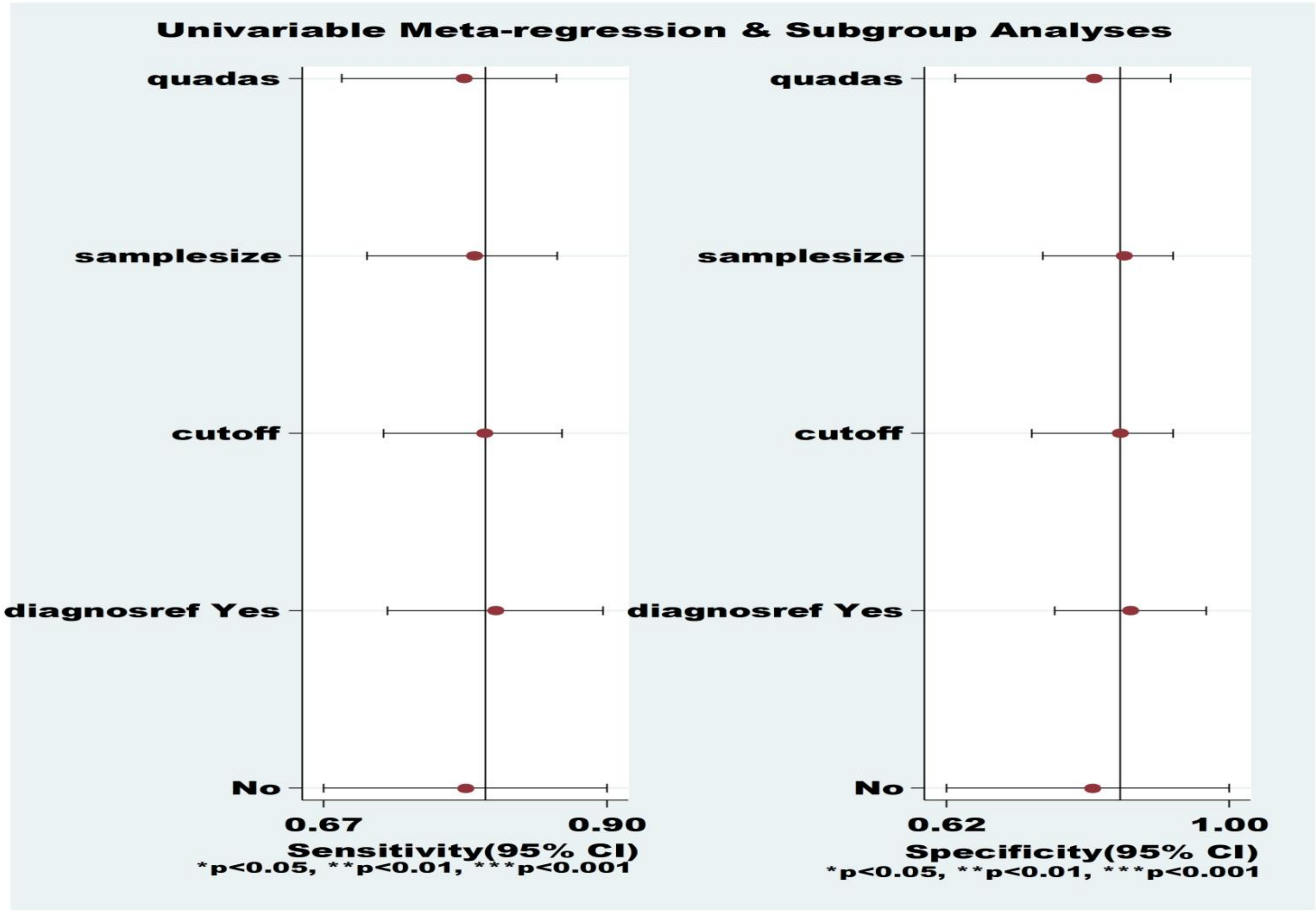

Figure S3. Meta regression

Accuracy of the serum intestinal fatty-acid-binding protein for diagnosis of acute intestinal ischemia: a meta-analysis

Da-Li Sun, Yun-Yun Cen, Shu-Min Li, Wei-Ming Li, Qi-Ping Lu, Peng-Yuan Xu

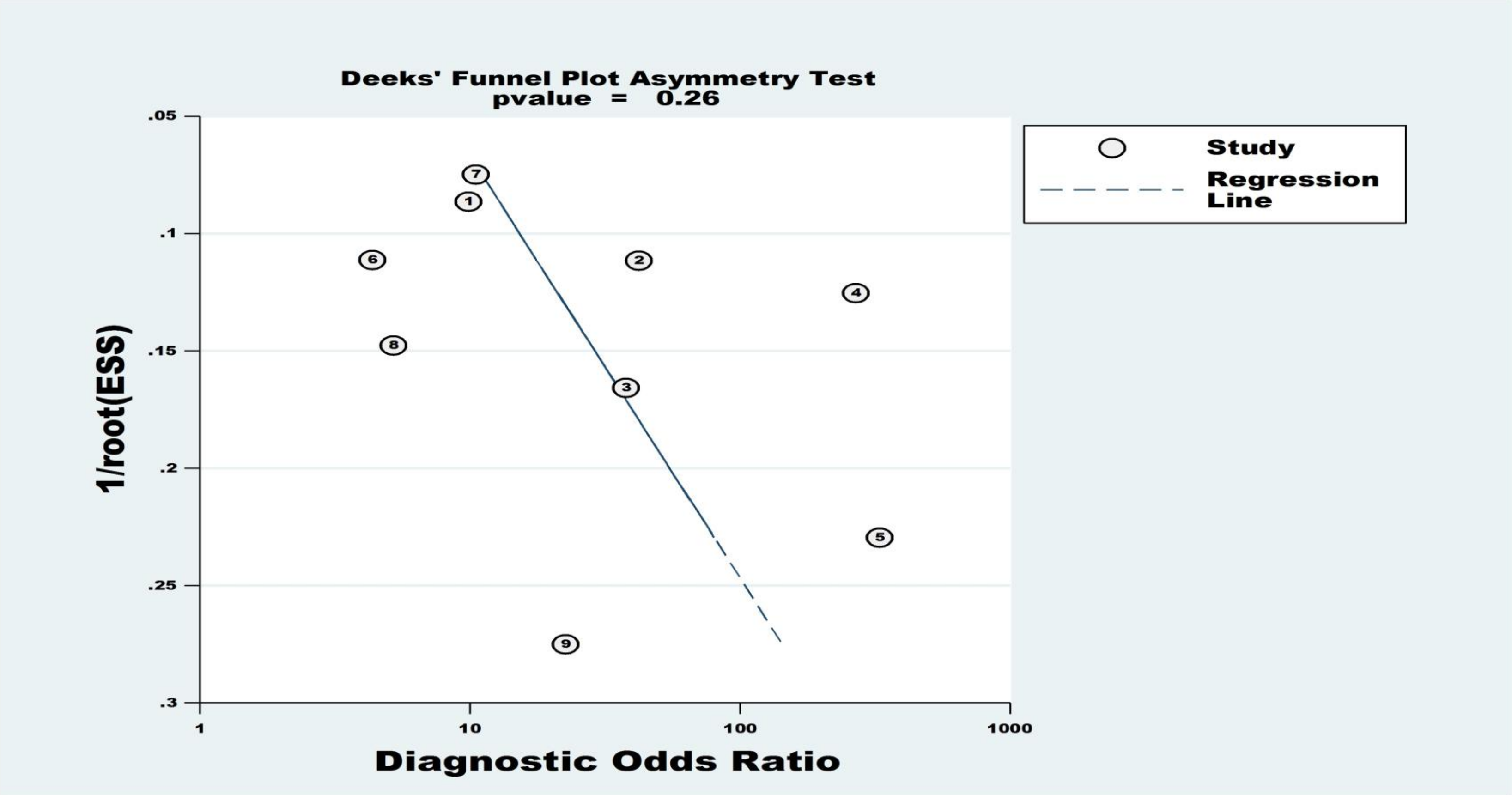

Figure S4. Deek's funnel plot showed no significant publication bias

Accuracy of the serum intestinal fatty-acid-binding protein for diagnosis of acute intestinal ischemia: a meta-analysis

Da-Li Sun, Yun-Yun Cen, Shu-Min Li, Wei-Ming Li, Qi-Ping Lu, Peng-Yuan Xu

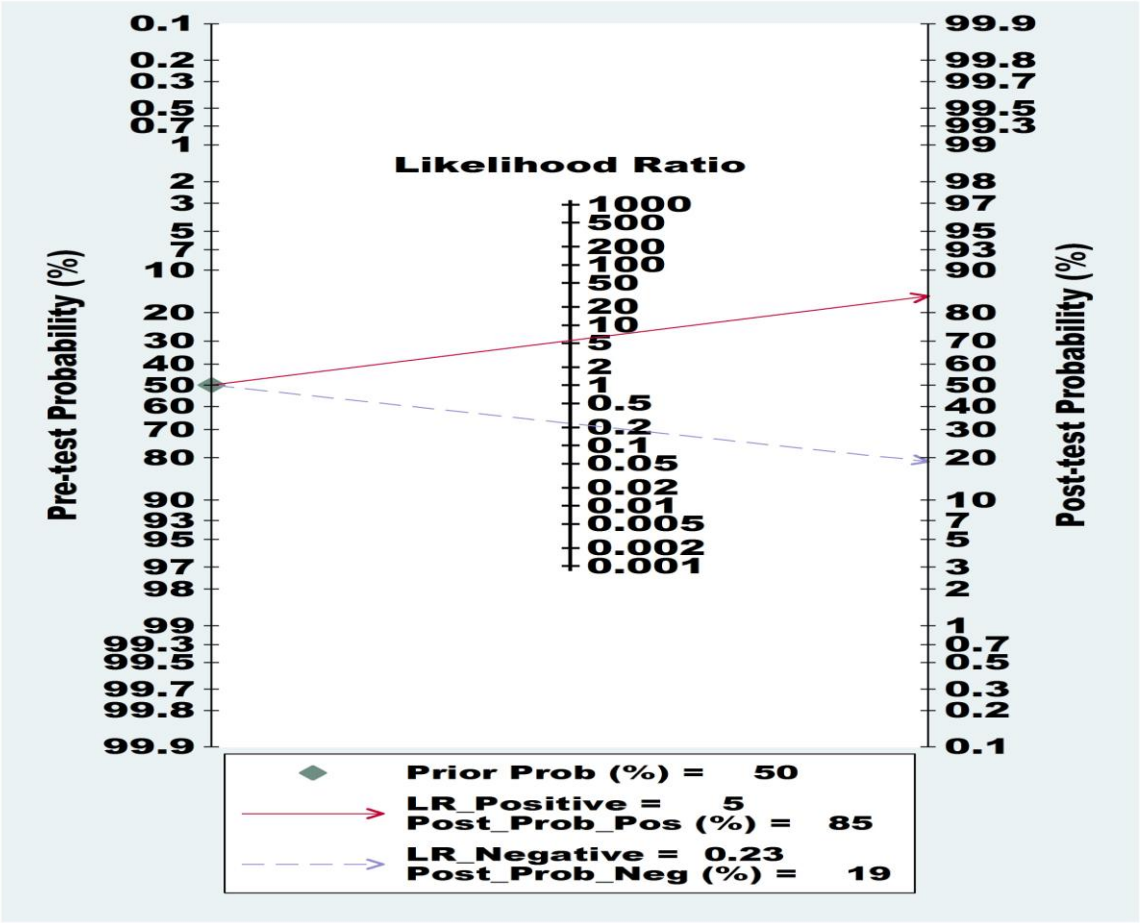

Figure S5. Fagan plot presents the clinical utility of I-FABP
